# In the screening of alternative insecticides to control *Aedes aegytpti* larvae 2-methylanthraquinone showed no genotoxicity and low toxicity to zebrafish (*Danio rerio*)

**DOI:** 10.1590/1678-4685-GMB-2021-0307

**Published:** 2022-02-28

**Authors:** Tathyana Benetis Piau, Maria Luiza Fascineli, Diego Sousa Moura, Lorena Carneiro Albernaz, Laila Salmen Espindola, Cesar Koppe Grisolia

**Affiliations:** 1 Universidade de Brasília, Instituto de Ciências Biológicas, Laboratório de Genética Toxicológica, Brasília, DF, Brazil.; 2 Universidade de Brasília, Faculdade de Ciências da Saúde, Laboratório de Farmacognosia, Brasília, DF, Brazil.; 3 Universidade Federal da Paraíba, Centro de Ciências da Saúde, Departamento de Morfologia, João Pessoa, RS, Brazil.

**Keywords:** Comet assay, micronucleus, larvicide, dengue control, quinones.

## Abstract

The threats posed by insecticide resistance to *Aedes aegypti* in the context of controlling dengue have led to an urgent search for an environmentally safer alternative chemical with more effective larvicidal properties. Among many molecules tested, 2-methylanthraquinone showed the lowest LC50 for *A. aegypti* in a previous study and the highest LC50 for zebrafish embryos. Embryos were exposed at concentrations of 1.0, 2.19, 4.78, 10.46, 22.87, 50.0 and 100.0 mg/L, and malformations and mortality were significantly observed only at the highest exposures of 50 and 100 mg/L after 96 h. Micronucleus test and comet assay in zebrafish adults were both negative after exposures at 6.25, 12.5, 25.0, 50.0 and 100.0 mg/L for 96 h. Several biochemical biomarkers were analyzed in adults, and 2-methylanthraquinone did not interfere with acetylcholinesterase activity. The lactate dehydrogenase activity was higher at concentrations of 25 and 100 mg/L. Glutathione-S-Transferase (GST) activities were tested in the gill and body (muscle tail). The gill was more sensitive than body for GST activity after exposure to 2-methylanthraquinone, showing the highest activities, and 2-methylanthraquinone showed low toxicity to a non-target organism.

## Introduction

Many arboviruses are transmitted by *Aedes aegypti*, such as dengue, chikungunya, yellow fever, and zika. Diseases caused by this mosquito vector are a public health problem in Latin American countries, especially in Brazil. Dengue in Brazil has the highest number of reported cases around the world (Rodriguez-Barraquer, 2011). The wide use of chemical insecticides based on pyrethroids, oganophosphates, carbamates and organochlorine in the combat of *A. aegypti* has caused insect resistance (WHO, 1998; Yang et al., 2020). In the search for alternative compounds with larvicidal effects to be used in the control of *A. aegypti,* natural products were included. 

Resistant populations of *A. aegypti* are expanding and invading many urbanized areas, in which they become well-established. Chemical control remains one of the main ways to combat *A. aegypti* populations worldwide. The quinones are a subset of the quinoid family, which also contains the quinone imines and cyclohexadienedione. They are commonly found in several natural products including plants, generated through the metabolism of aromatic compounds. The quinones present different toxicological properties, including cytotoxicity, neurotoxicity, hepatotoxicity, immunotoxicity, genotoxicity and carcinogenicity (Bolton and Dunlap, 2017). 

Different types of quinones are involved in DNA damage, including depurination, DNA-adducts, base oxidation such as 8-Oxo-2’-deoxyguanosine, of which DNA oxidative damage is the most common mutagenic effect. Quinones cause oxidative stress through the consumption of reducing equivalents, including GST - Glutathione-S-Transferase and NAD(P)H (Gant et al., 1988). Chemical reactivity is based on oxide-reduction reactions, producing reactive oxygen species (ROS) and therefore DNA strand breaks and DNA crosslinks. Their precise mechanism of action is not completely understood, but DNA is also a direct target by alkylation or intercalation. (El-Najjar, 2011; Bolton and Dunlap, 2017).

The threats posed by chemical resistance demand an urgent search for an environmentally safer alternative method with more effective larvicidal properties. At the same time, the screening of new larvicides against *A. aegypti* must take into account low toxicity to non-target organisms. Since they are used in water, studies showing ecotoxicological information should be carried out in aquatic organisms. We have been screening many quinone-based compounds with larvicidal properties against *A. aegypti*. Among many molecules tested, 2-methylanthraquinone showed efficacy to control *A. aegypti* larvae, with low LC50 (24 h) = 1.10 µg/ml and LC50 (48 h) = 0.75 µg/ml (Silva *et al*, 2020). In contrast, it showed a high LC50 > 100 mg/L for zebrafish embryo, as determined in this study. Therefore, based on previous results, we used zebrafish (*Danio rerio*) embryos, larvae and adults for evaluations of genotoxicity and toxicity of 2-methylanthraquinone through different endpoints.

## Material and Methods

### 2-methylanthraquinone from (Thermo-Fisher), with purity of 97%, Figure 1.

**Figure 1 -:**
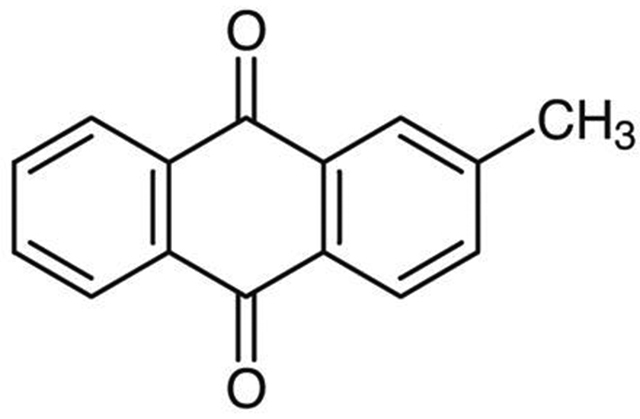
2-methylanthraquinone, CAS number 84-54-8, molecular formula C_15_H_10_O_2_, molecular weight 222.24.

### Test organisms

Zebrafish adults were raised in a facility with reverse osmosis and activated carbon filtered water (ZebTec - Tecniplast, Italy) with a photoperiod cycle of 12:12 h (light:dark) at the Department of Genetics and Morphology, University of Brasília (Brazil). The water parameters were controlled: temperature at 27.0 ± 1 ºC, conductivity at 650 ± 100 µS/cm, pH at 7.0 ± 0.5 and dissolved oxygen ≥ 95% saturation. Zebrafish embryos were obtained by breeding of fish in the Ispawn breeding system (Tecniplasty). The day prior to breeding, males and females were sequentially added to the system and kept separated by a divider, in a proportion of two males to one female. Early in the morning, the divider was removed and the spawning platform was lifted to initiate the spawning. The embryos were collected immediately after natural mating, rinsed in water, and checked under a stereomicroscope (Stemi 2000, Zeiss). The unfertilized eggs (< 20%) and those with cleavage irregularities or injuries were discarded. This study was previously approved by the ethics committee at the University of Brasilia (protocol n. 113/2019).

### Fish embryo toxicity test

The fish embryo toxicity (FET) test was based on the OECD guideline Protocol 236. Zebrafish embryos were exposed to 1.0, 2.19, 4.78, 10.46, 22.87, 50.0 and 100.0 mg/L, dissolved in DMSO at 0.05%, a control group, and a DMSO (1%) group for 96 h. These exposure concentrations were chosen based on the previously determined LC50 in a pilot test in our laboratory. The test was performed using 60 embryos per treatment, divided in 3 replicates, selected and distributed in 96-well microplates in the climate chamber (SL-24 Solab Científica, Brazil). The test was initiated immediately after fertilization. Embryos and larvae were observed daily under a stereomicroscope and before hatching, and the following parameters were evaluated: egg coagulation, otolith formation, general delay in development, eye and tail pigmentation, somite formation, heartbeat, oedemas, detachment of the tail-bud from the yolk sac, yolk sac absorption and hatching. After hatching, spine malformation and equilibrium were also evaluated. All parameters were assessed and quantified as observed or not observed.

### Fish adult toxicity test: comet assay and micronucleus test

Comet assay: this was performed following the alkaline conditions (pH > 13) described by Singh et al., (1988) with modifications. 100 µl of cell-suspension sampled from peripheral erythrocytes was mixed in 120 µl of low melting agarose at 37 °C. Then, 200 µl of erythrocyte-agarose suspension were placed onto a frosted slide pre-coated with standard agarose (1.5%), and covered with a coverslip. The slides were stained with ethidium bromide (20 μg/mL) and analyzed using a fluorescence microscope (Axioskop 2, Zeiss, at 400× magnification), fitted with a 510-560 nm filter and a 590 nm blocking filter; and the Comet IV Lite v4.3 system image analysis software (Perceptive Instruments, Suffolk, UK). The percentage of DNA in the tail (% DNA in tail) was measured in 100 nucleoids of each fish (Hartmann et al., 2003). Groups of 8 fish at 6 months old were exposed for 96 h at concentrations of 6.25, 12.5, 25.0, 50.0 and 100.0 mg/L of 2-methylanthraquinone dissolved in water and Dimethyl-suphoxide (DMSO) at 0.005%), plus negative, positive (H_2_O_2_ at 1%), and DMSO controls. 50 µl of peripheral blood from each fish were used In the post-exposure test (recovery), fish that were exposed to 6.25, 25.0 and 100.0 mg/L, negative and DMSO controls were maintained for a further 96 h in clean water.

Micronucleus (MN) and nuclear abnormalities (NAs) tests: peripheral blood samples were smeared on clean glass slides, dried at room temperature, fixed in methanol for 7 min and stained by Giemsa (5%). The slides were evaluated under a blind code; 3000 erythrocytes were microscopically scored for each sample at 1,000X of magnification (1,500 erythrocytes per slide). The criteria for the identification of micronucleated erythrocytes of fish were: area smaller than one-third of the main nucleus; no connection with the main nucleus; no refraction and same color and color intensity as in the main nucleus. Erythrocytes were also scored to classify nuclear abnormalities such as BB -blebbed, LB - lobed, NT - notched, CV - cell vacuoles, BN - bilobed nucleus and nuclear bud NB (Carrasco et al., 1990; Al Sabti and Metcalfe, 1995). Following the comet assay, the micronucleus was also evaluated in the post-exposure (recovery) of fish that were exposed to 6.25, 25.0 and 100.0 mg/L, negative and DMSO controls; all of these were maintained for a further 96 h in clean water. The blood samples were obtained through cardiac puncture after anesthesia with tricaine. Blood from the same fish was used for MN test and comet assay. 

### Biochemical biomarkers in adult zebrafish

For AChE activity, we used samples from tail muscle (body) and head. The tail muscle was dissected. The head was used without dissecting (entire). For LDH, we used only samples from the tail muscle dissected (isolated). Acetylcholinesterase (AChE) activity was determined using acetylthiocholine (ASCh) and propionylthiocoline (PSCh) as substrates, measuring the conjugation product between thiocoline (result of the degradation of ASCh or PSCh) and 5,5-dithiobis-2-nitrobenzoic acid (DTNB) (absorbance increase) at 414 nm, every 20 s, for 05 min, according to the method previously described by Ellman et al., (1961). For the enzymatic determination, 40 μl of Pos-mitochondrial supernadant (PMS) and 250 μl of reaction mixture (acetylcholine and propionylcholine (75 mM) and DTNB (10 mM)) in K-phosphate buffer (0.1 M, pH 7.2) were used. AChE activities were expressed as micromoles of substrate hydrolyzed per minute per mg of protein (U). The protein concentration of samples was quantified using the Bradford Method at 595 nm and γ-globulin as standard (Bradford, 1976). All reactions were performed spectrophotometrically (SpectraMax M2 microplate reader) in quadruplicate. Adults (6 months old) were exposed for 96 h to 6.25, 12.5, 25.0, 50.0 and 100.0 mg/L dissolved in DMSO 0.005%, plus DMSO negative controls. In the recovery evaluation, fish that were previously exposed to 6.25, 25.0, 100.0 mg/L, negative and DMSO controls were maintained for a further 96 h in clean water. Samples of the head and body were obtained and immediately frozen in liquid nitrogen and stored at −80 °C until the day of analysis. For AChE enzymatic activity measurement, samples were defrosted on ice, homogenized using a sonicator (Ultrasonic Cleaner 2840D Odontobras), and centrifuged refrigerated (4 °C) for 20 min at 10,000 g. The resulting post-mitochondrial supernatant was isolated, and 40 μl of each sample was pipetted in 96-well microplates for enzymatic determinations performed spectrophotometrically (Multiskan FC, Thermo, USA) in quadruplicate.

Lactate Dehydrogenase (LDH) activity was determined using a pyruvate as substrate and measuring the reduction of Pyruvate and the oxidation of NADH at 340 nm, every 40 s, for 5 min. Activity determinations were carried out using 40 ml of PMS, 260 ml of reaction mixture (NADH (0.24 mM) and pyruvate (10 mM)) Tris-NaCl buffer (0.1 M, pH 7.2) (Vassault, 1983).

Glutathione-S-Transferase (GST) activity was determined from isolated gill and tail muscle, using a thioether as substrate and measuring the conjugation product between glutathione and 1-Chloro-2,4-dinitrobenzene (CDNB) (absorbance increase) at 340 nm, every 40 s, for 5 min, according to the method of Habig et al., (1981). Activity determinations were carried out using 50 μl of PMS, 250 μl of reaction mixture (glutathione (10 mM) and CDNB (10 mM)) in K-phosphate buffer (0.1 M, pH 6.5).

### Statistical analysis

All statistical analysis was performed using SigmaPlot (version 12.5). One Way ANOVA was done to detect differences between groups for normally distributed datasets. Data that did not pass the Shapiro-Wilk normality test and the Brown-Forsythe homogeneity of variance test were tested by Kruskal-Wallis. Dunnett’s or Dunn’s test (for parametric or non-parametric tests, respectively) were used to detect significant differences between the tested concentrations and controls (p<0.05). Lethal concentrations (LC50) were calculated using logistic Weibull, or sigmoid function. For analysis of biochemical biomarkers, comparing exposure tests with clearance period (recovery test), the Student t test for paired samples was used.

## Results

**Figure 2 -:**
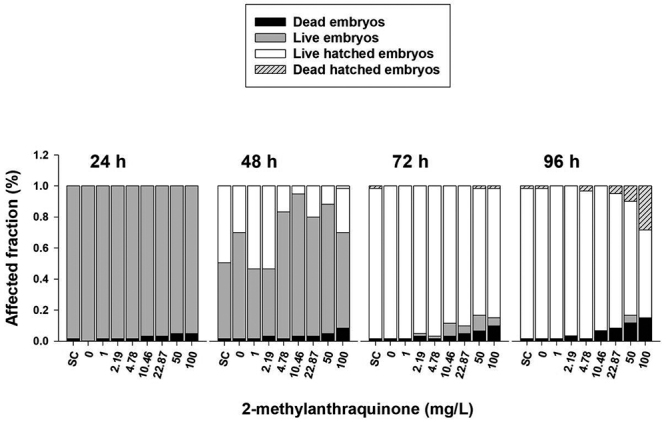
An overview FET-test showing low mortality and embryo malformation rates of 2-methylanthraquinone to zebrafish.

**Figure 3 -:**
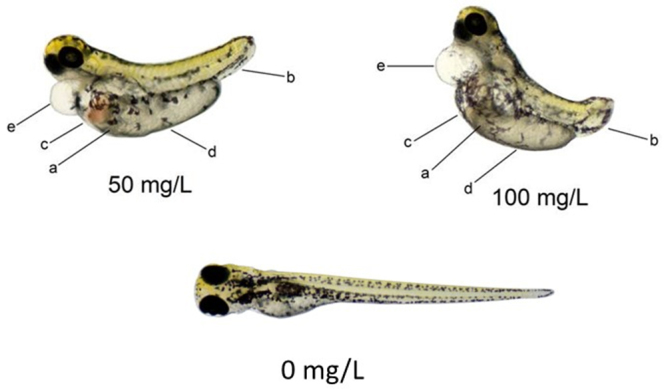
The main embryo alterations found at exposures of 50 and 100 mg/L for 96 h. 0 mg/L control. a. yolk sac edema, b. tail malformation, c. stasis, d. yolk sac malformation, e. pericardic edema.

For embryotoxicity, Figure 2 shows an overview of the mortality rate. The LC50 (96 h) of 2-methylanthraquinone is above 100 mg/L. A significant mortality rate was observed only at the highest concentrations of 50 and 100 mg/L after 96 h of exposure. At these concentration levels some embryo malformations in tail, yolk sac and cardiac edema were also observed (P<0.05). The presence of dark-purple spots in the abdomen around the liver region in development can represent hepatotoxicity causing blood stasis (Figure 3c). Due to zebrafish embryo transparency, these dark-purple spots near the liver were easily seen only at the highest exposure levels (Figure 3c). The frequencies of such alterations were directly associated with exposure levels and exposure time, mainly with 50 and 100 mg/L at 96 h. 

**Figure 4 -:**
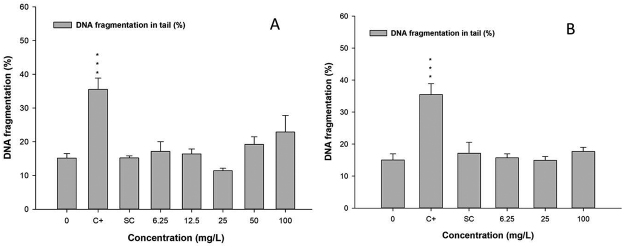
Comet assay, A - exposure test, B - post-exposure test, C+ positive control, SC - solvent control (DMSO). No DNA fragmentation induction occurred in both cases (P>0.05). DNA fragmentation was observed in C+ group (**<0.01 and ***<0.001) when compared with control.

In the genotoxic studies in the zebrafish adults, the tested concentrations were based on the LC50 obtained previously and also following OECD protocol n. 203 - Fish, Acute Toxicity Test. In the results of the comet assay analyzing tail length, 2-methylanthraquinone did not show any genotoxicity. The recovery tests evaluating post-exposure effects were also negative (Figure 4). Evaluations of micronucleus and nuclear abnormalities were carried out in peripheral erythrocytes. Considering exposure and post-exposure tests, these results were also negative (Table 1). 

**Table 1 -:** MN - micronucleus, NBUD - nuclear bud, BN - bilobed nucleus, BB - blebbed, LB - lobed, NT - notched, CV - cell vacuoles. In the case of both the exposures and post-exposures, no genotoxicity was found compared with controls (P > 0.05).

Treatment	Concentration (mg/L)	MN	NBUD	BN	BB	LB	NT	VC
Exposure	0	0.1 ± 0.1	0.2 ± 0.3	0.2 ± 0.2	0.1 ± 0.1	0.2 ± 0.3	0.2 ± 0.3	0.2 ± 0.5
	SC	0.1 ± 0.1	0.2 ± 0.5	0	0.5 ± 0.7	0.2 ± 0.4	0.1 ± 0.1	0.1 ± 0.1
	6.25	0	0.1 ± 0.1	0.1 ± 0.4	0.3 ± 0.3	0.2 ± 0.3	0.3 ± 0.5	0
	12.5	0 ± 0.1	0.2 ± 0.3	0	0.7 ± 0.8	0.2 ± 02	0.2 ± 0.2	0.1 ± 0.3
	25	0 ± 0.1	0.1 ± 0.2	0.1 ± 0.1	0.2 ± 0.3	0.3 ± 0.3	0.3 ± 0.4	0.1 ± 0.3
	50	0	0	0 ± 0.1	0.3 ± 0.3	0.1 ± 0.1	0.3 ± 0.6	0
	100	0.2 ± 0.3	0.2 ± 0.3	0.1 ± 0.3	0.1 ± 0.2	0.1 ± 0.1	0.3 ± 0.2	0
Post-exposure	0	0.2 ± 0.3	0.2 ± 0.3	0	0.2 ± 0.2	0.3 ± 0.4	0.4 ± 0.5	0.4 ± 0.8
	SC	0.2 ± 0.3	0	0	0.1 ± 0.1	0.1 ± 0.1	0.1 ± 0.2	0.5 ± 0.7
	6.25	0.1 ± 0.2	0 ± 0.1	0.2 ± 0.4	0.4 ± 0.7	0.2 ± 0.2	0.2 ± 0.3	0
	25	0 ± 0.1	0.1 ± 0.2	0.1 ± 0.1	0.2 ± 0.3	0.3 ± 0.3	0.3 ± 0.4	0.1 ± 0.3
	100	0.5 ± 0.7	0.4 ± 0.4	0	0.2 ± 0.4	0.1 ± 0.1	0.2 ± 0.2	0

**Figure 5 -:**
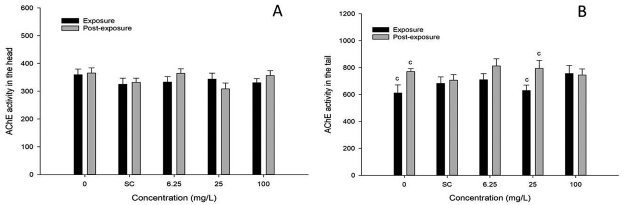
A - AChE activity in zebrafish head (P>0.05), B - AChE activity in zebrafish tail, c - significant difference between exposure and post-exposure (P <0.05).

The results of the measurement of AChE activities in the zebrafish adults head and tail (Figure 5). There were no differences compared with controls. In the recovery test (clearance), the results remained constant. 2-methylanthraquinone did not interfere with this neuronal transmitter. Only one significant difference was observed at 25 mg/L in the post-exposure test of tail samples, which could be a casual response, since the same variation occurred in the controls.

**Figure 6 -:**
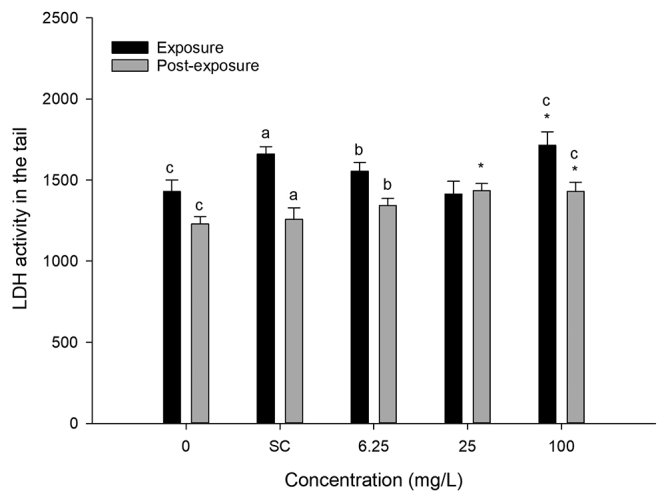
Measurement of LDH activity in zebrafish tail, comparing results of exposures with post-exposures. sc - solvent control, * - statistically significant compared with control (P<0.05). a (P<0.001), b (P<0.01), c (P<0.05). a, b and c.

The LDH activity resulted in more effective action at concentrations of 25 and 100 mg/L of 2-methylanthraquinone, where the presence of the compound caused more cell injuries. The recovery test demonstrates that in the absence of exposure there were decreased LDH activities, and clearance (Figure 6).

**Figure 7 -:**
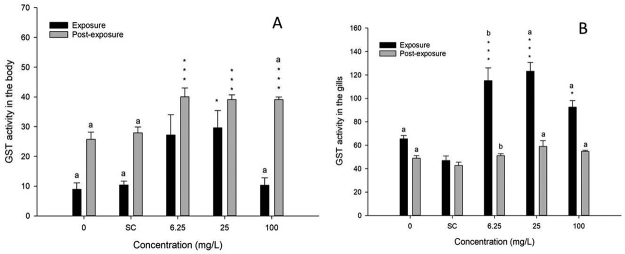
GST **A** - activity in tail muscle (body) showing the results of exposure test and the recovery period of clearance. **B** - in gills showing the difference between exposure test and recovery period. * (P<0.05), *** P<0,001), a (P<0.001), b (P<0.01). a and b - comparing exposure with post-exposure.

In the GST tests, more evident effects of exposure to 2-methylanthraquinone were observed among the other biochemical markers studied (Figure 7). The results showed that gills were more responsive to GST activity than tails. In gills, exposures at 6.25, 25.0 and 100.0 mg/L were quite different from controls (Figure 7B, P<0.001). In contrast, in tails such differences were not observed (Figure 7A, P>0.05). In the recovery period of clearance, the GST activity in the tail remained high, but not statistically different from the exposure test (P = 0.135). In the gill, the post-exposure period showed statistically significant decreased GST activity (Figure 7B, P<0.001), which means that in gills the clearance was more effective.

## Discussion

Due to their wide occurrence in nature, anthraquinones are involved in many different biological processes ranging from antioxidant to pro-oxidant properties. The knowledge of the chemical reactivity of anthraquinones helps to understand their toxicities (Bolton et al., 2000). It is no longer necessary to show that embryo development is the most sensitive stage in the life cycle. That is why FET - OECD 236 using zebrafish was adopted by REACH (Registration, Evaluation, Authorizations and Restriction of Chemicals) in the evaluation of chemicals for regulatory authorities in the European Union. This guideline recommends an exposure concentration limit at 100 mg/L or a limit based on the solubility of the tested compound. LC50>100 mg/L means low toxicity, as well as low risk to aquatic life, since the LC50 found in *A. aegypti* larvae is 1.1 mg/L (Silva et al., 2020). Evidence of zebrafish embryotoxicity was observed only at the highest exposures of 50 and 100 mg/L. At these exposure-concentrations, toxicity became more evident, showing a significant number of alterations, mainly edema in the yolk sac, pericardial edema, blood stasis in the abdomen, and tail malformations. 

The dark-purple spots found in the fish abdomen can be caused by blood stasis due to liver injury. The liver is the organ most susceptible to blood stasis, which may be caused by disorders that affect circulation (Neeb, 2007). Liver inflammation can cause disorders in microvascular circulation, retaining blood and leading to stasis (Xiao-Hong, *et al.,* 2020; Yang *et al.,* 2021). As liver histopathology was not carried out, exposures to 2-methylanthraquinone at toxic levels are suspected of drug-induced liver injury. Emodin (1,3,8-trihydroxy-6-methyl anthraquinone), a herbal medicine, causes hepatotoxicity due to inflammatory stress and liver injury (Tu *et al.,* 2015). It is improbable that a spray using this proposed new larvicide in the control of *A.aegypti* will reach these concentrations in the natural environment. Thus, 2-methylanthraquinone demonstrates high lethality for the target organism and low lethality for the non-target organism. 

Regarding genotoxic activities, the active redox capability of the anthraquinones, producing reactive ROS, suggests their major action on DNA and chromosomes. The presence of hydroxyl groups in the quinone molecules is directly associated with their reactivity with DNA. The hydroxyanthraquinones, purpurin and emodin, with 2 hydroxy groups, showed mutagenicity in *Samonella* (Ames test) and gene mutation in HPRT locus of V79 cells, whereas the anthraquinones, danthron and alizarin, without hydroxyl groups were devoid of mutagenicity (Westendorf et al., 1990; Tu et. al., 2015; Jinlan et.al., 2021). However, 2-methylanthraquinone does not present hydroxyl groups, which could explain the absence of genotoxicity. In this case, neither the micronucleus test nor the comet assay was positive. The DNA crosslinks are not detected under standard comet assay, because crosslinks prevent separation of strands, resulting less DNA migration (Merk et al., 2000). As pointed out in the study of Bolton and Dunlap (2017), that some quinones can cause DNA crosslinks, and then some false negative could be occurred in this study.

Considering biochemical markers, AChE is an extensively studied enzyme of importance for synaptic integrity, neurite outgrowth, and neurodevelopment (Delwing et al., 2005). AChE can be used as an *in vivo* neurotoxicity marker in aquatic vertebrate species for monitoring environmental quality, where the prolonged inhibition in the synaptic cleft leads to muscular contraction and toxic effects (Delwing *et al.,* 2005). In *Cyprinus carpio* exposed to carbamate pesticides, AChE was strongly inhibited in the muscle (Lavado et al., 2006). Zebrafish have showed high sensitivity to the well-known AChE inhibitors, organophosphate and carbamate pesticides (Küster, 2005). For all tested concentrations there were no changes in the AChE levels for head and tail samples, compared with respective controls. 

Lactate dehydrogenase (LDH) plays an important role in energy metabolism, being found in almost all tissues. When tissues are damaged, LDH is released into the bloodstream, which means some cell injury has occurred. LDH is used to measure cytotoxicity caused by pesticides and drugs, leading to the destruction of cell membranes or lysis (Bopp and Lettieri, 2008). Analyzing our results, there were increases in the LDH levels only at the highest exposure of 100 mg/L, as well as at 25 and 100 mg/L in the recovery period, which means that 2-methylanthraquinone can be cytotoxic in higher concentrations, and this effect remains after exposures.

GST, a phase II conjugation enzyme, is known for its ability to detoxify xenobiotics. The GST family in zebrafish is well characterized, revealing high expression in kidney, brain, liver and intestine (Glisic et al., 2015). The activity of GST was measured in the gill and tail of zebrafish adults after exposure to 2-methylanthraquinone. In this study, the presence of the xenobiotic in water increased the GST activity in the zebrafish gills and tail. However, in the exposed fish, gills showed the highest GST activities. In the recovery test of clearance, these values decreased in gills, demonstrating that there really was detoxification. The increased GST activity in the tail was more discreet, remaining in the recovery period, showing that the detoxification process in the tail may be occurring slowly. It makes sense, because the gills are in direct contact with xenobiotics in water and metabolize faster. In conclusion, 2-methylanthraquinone as appeared to be a good candidate for an alternative insecticide in dengue control, but more studies on its potential risks to other non-target organisms in the aquatic ecosystem should be carried out.
